# Personal Mention

**Published:** 1916-01

**Authors:** 


### Personal Mention.

The many friends and admirers of Dr. John S. Turner, of Dallas, will be delighted to know of his return from the Cumberland Mountains, where he has been recuperating for the last two and a half months.

Dr. W. L. Crosthwait, of Waco, has just returned from Chicago and Rochester, where he has been doing some post-graduate work.

The case against six Fort Worth doctors for giving alleged illegal prescriptions for morphine and cocaine will come to trial the first of January.

Dr. W. L. Culpepper, formerly of Temple, Texas, has been appointed to take permanent charge of the laboratory in the bureau of health of Syracuse, New York.

Dr. S. S. Munger has been appointed city health officer of Marlin.

Dr. C. A. Traweek, of Matador, recently had his handsome home destroyed by fire.

Dr. J. H. Spivy has been appointed county health officer of Henderson county.

Dr. Frank Paschal, of New York, has obtained the appointment to work in the hospital in Berlin. This is a very distinguished honor for so young a man. Out of one hundred applicants only three received appointment, Dr. Paschal being one of the three. He is the son of Dr. Frank Paschal, of San Antonio, and is truly proving himself the worthy son of a noble sire.

Dr. and Mrs. J. H. Eastland, of Mineral Wells, have just returned from a tour of the Pacific Coast from Tia Juona, Mexico, to Vancouver, B. C.

Dr. Albert J. Caldwell announces the removal of his office from Corpus Christi, Texas, to suite 513 Gibbs Building, San Antonio, Texas. Practice limited to eye, ear, nose and throat.

